# Extended-spectrum beta-lactamase production and multi-drug resistance among *Enterobacteriaceae* isolated in Addis Ababa, Ethiopia

**DOI:** 10.1186/s13756-019-0488-4

**Published:** 2019-02-15

**Authors:** Dejenie Shiferaw Teklu, Abebe Aseffa Negeri, Melese Hailu Legese, Tesfaye Legesse Bedada, Hiwot Ketema Woldemariam, Kassu Desta Tullu

**Affiliations:** 1 Department of Clinical Bacteriology and Mycology National Reference Laboratory, Ethiopian Public Health Institute, Addis Ababa, Ethiopia; 2 Department of Medical Laboratory Sciences, College of Health Sciences, Addis Ababa University, Addis Ababa, Ethiopia; 3 Department of Clinical Bacteriology and Mycology National Reference Laboratory, Ethiopian Public Health Institute, Addis Ababa, Ethiopia; 4 Department of Public Health Microbiology, Ethiopian Public Health Institute, Addis Ababa, Ethiopia; 5 Department of Virology, Ethiopian Public Health Institute, P. O. Box, 1242 Addis Ababa, Ethiopia

**Keywords:** ESBLs, MDR, *Enterobacteriaceae*, Clinical specimens, Addis Ababa, Ethiopia

## Abstract

**Background:**

The global emergence and spread of extended-spectrum beta-lactamases (ESBLs) producing *Enterobacteriaceae* have been threatening the ability to treat an infection. Hence, this study aimed to determine the prevalence of ESBL-producing and multi-drug resistance (MDR) *Enterobacteriaceae* (ESBLs-E) from different clinical specimens in Addis Ababa, Ethiopia.

**Methods:**

A cross-sectional study was conducted from January 1 to May 30, 2017. A total of 426 *Enterobacteriaceae* isolates were identified from clinical specimens. The isolates were collected from four laboratories. Antimicrobial susceptibility testing was performed using the Kirby-Bauer disk diffusion method on Muller Hinton agar (MHA). All *Enterobacteriaceae* were screened for ESBLs production using cefotaxime and ceftazidime as per CLSI guideline. Each ESBL screening positive *Enterobacteriaceae* were confirmed by a combination disk test (CDT). Data were entered and analyzed by using SPSS version-20.

**Result:**

The most frequent *Enterobacteriaceae* were *E. coli* 228 (53.5%) and *K. pneumoniae* 103 (24.1%). The magnitude of ESBLs-E was 57.7% (246/426). The highest frequencies of ESBLs-E were observed in blood specimesns (84.4%) and the highest ESBLs production was observed in *K. pneumoniae* (85.4%). The highest resistance level was seen to sulfamethoxazole-trimethoprim (77.0%), amoxicillin with clavulanic acid (71.6%), cefotaxime (62.2%), cefepime (60.3%) and ceftazidime (60.8%). The overall magnitude of multi-drug resistance (MDR) level was 68.3%. Of ESBLs-E, 96.3% of them were MDR (*P* < 0.001).

**Conclusion:**

There was a high prevalence of ESBLs-E and MDR isolate in Addis Ababa. Most of ESBLs-E was isolated primarily in blood and urine. The highest ESBLs production was observed among *K. pneumoniae*. Hence, strong infection control strategies must be implemented in hospital settings of the country.

## Introduction

*Enterobacteriaceae* are Gram-negative, facultative anaerobes, and non-sporing bacilli. These bacteria have become one of the most important causes of nosocomial and community-acquired infections. They can cause urinary tract, respiratory tract, and bloodstream and wound infections. Increasing rates of antimicrobial resistance have become a worldwide problem predominantly caused by Gram-negative bacteria, the *Enterobacteriaceae* [1, 2]*.*

Beta-lactam drugs such as extended-spectrum penicillins, cephalosporins, monobactams, carbapenems, fluoroquinolones (e.g. ciprofloxacin) and aminoglycosides (e.g. gentamicin) are among the most prescribed antibiotics to treat infections caused by *Enterobacteriaceae.* The widespread use of beta-lactam antibiotics has caused the expansion of resistant *Enterobacteriaceae*. The most important mechanism of resistance to beta-lactam antibiotics involves the production of beta-lactamases (especially extended-spectrum beta-lactamases) that inactivate beta-lactam antibiotics and this continue to be the prominent cause of β-lactam antibiotics resistance among *Enterobacteriaceae* worldwide. ESBL-producing *Enterobacteriaceae* are important members of antibiotic-resistant bacteria that cause hospital and community-acquired infections [3, 4].

ESBL is an enzyme that is produced by bacteria to become resistant to extended-spectrum penicillin, cephalosporins, and monobactams except for cephamycins and carbapenems. It is also inhibited by beta-lactamase inhibitors like clavulanic acid. A worrisome increasing trend has been reported on the development of resistance to extended-spectrum cephalosporins caused by ESBL producing *Enterobacteriaceae* [2, 3, 5]. Among *Enterobacteriaceae*, ESBLs have been found mostly in *Klebsiella* spp. and *E. coli* as well as in other *Enterobacteriaceae* families such as *Enterobacter spps.*, *Proteus spps*, *Citrobacter spps*, *Morganella spps*, *Providencia spps.*, *Salmonella spp.*, and *Serratia spps* [6–8]*.*

Being plasmid mediated, ESBL is easily transmitted among members of *Enterobacteriaceae.* The dissemination of this resistance applies not only to beta-lactams but also to other commonly used antibiotics such as fluoroquinolones, aminoglycosides, and sulphonamides [9, 10]. Consequently, many patients need the ‘last resort’ antibiotics treatment such as carbapenems [2, 11]. Again the use of carbapenems has led to the rapid selection of carbapenem-resistant *Enterobacteriaceae* [12]. Only a few antibiotics (e.g. carbapenems, colistin, tigecycline) are available to treat infection caused by ESBL-producing bacteria, although the in vivo efficacy and/or toxicity of these drugs is not well well-known [13, 14].

Assessing ESBL producing *Enterobacteriaceae* in the local scenario is necessary to understand the epidemiology and the disease burden as well as to design and implement hospital infection control strategies to prevent the further occurrence and spread of such bacteria. However, little is known about the magnitude of ESBL producing *Enterobacteriaceae* in Addis Ababa, Ethiopia. Moreover, to the best of our knowledge, almost all clinical bacteriology laboratories in Ethiopia do not perform ESBL tests. Hence, this study aimed to determine the prevalence of ESBL producing and MDR *Enterobacteriaceae* in different clinical specimens in Addis Ababa, Ethiopia.

## Methods

### Study setting

A laboratory-based cross-sectional study was conducted from January to May 2017 at the Ethiopian Public Health Institute (EPHI) Clinical Bacteriology and Mycology National Reference Laboratory in Addis Ababa. This laboratory is Ethiopia’s main referral laboratory and is accredited by the Ethiopian National Accreditation Office (ENAO). The *Enterobacteriaceae* isolates used for this study were collected from four microbiology laboratories: EPHI clinical bacteriology laboratory, International Clinical Laboratories (ICL), Tikur Anbessa Specialized Hospital (TASH), and Yekatit 12 Medical College Hospital Microbiology Laboratory. The isolates were collected using a convenient sampling technique. All consecutive *Enterobacteriaceae* isolated from clinical specimens in the selected bacteriology laboratories were included in the study. Demographic characteristics of the patients were recorded using a pre-developed worksheet. The isolates were collected using *Tryptose Soy Broth* (TSB) (Oxoid Ltd., Basingstoke, United Kingdom) containing 20% glycerol and temporarily stored at − 20 °C in the respective laboratory. Within a week the isolates were transported to the EPHI clinical bacteriological laboratory using a cold box with ice.

### Culture and identification

The isolates preserved at − 70 °C were recovered by re-suspension of the stored isolate in *Tryptose Soy Broth* (Oxoid Ltd., Basingstoke, United Kingdom)*.* After a few hours, the isolates were inoculated and incubated on MacConkey agar (Oxoid Ltd., Basingstoke, United Kingdom) at 37 °C for 18–24 h. After incubation, the colony was characterized by colony appearance, Gram stain, and biochemical tests. The isolates were identified by standard microbiological laboratory methods [15]. Antibiotic susceptibility and ESBLs confirmatory tests were done using the pure isolate sub-cultured on to 5% sheep blood agar (HiMEDIA Laboratories Pvt. Ltd., Mumbai, India).

### Preparation of clavulanate stock solution

For the combination disk test CDT method, the combined disks (Ceftazidime-clavulanate (30 μg/10 μg), and cefotaxime-clavulanate (30 μg/10 μg) disks) were prepared from in-house made clavulanate solution according to CLSI guideline [16]. From potassium clavulanate analytical standard powder (Sigma-Aldrich Corp, St. Louis, MO USA) stock solution of clavulanate at 1000 μg/ml was prepared, aliquoted, and stored at − 70 °C. When we were ready to perform CDT (each day of testing), 10 μL of clavulanate solution was added to ceftazidime (30 μg) and cefotaxime (30 μg) disks (Abtek Biologicals Ltd., Liverpool, United Kingdom) and we allowed about 30 min for the clavulanate to absorb, and the disks to be dry enough for application. The combined disks were used immediately (within 30 min) after they had dried.

### Antibiotic susceptibility testing

Antimicrobial susceptibility testing was carried out by the Kirby-Bauer disc diffusion method and the results were expressed as susceptible, intermediate or resistant according to CLSI guideline [16]. After preparation of 0.5McFarland turbidity inoculums, Muller-Hinton Agar (MHA) (Oxoid LTD, Basingstoke, Hampshire, United England) plates were inoculated and antimicrobial discs were applied to the plate. The antibiotic discs used in this study were amoxicillin-clavulanic acid (AMC: 20/10 μg), cefotaxime (CTX: 30 μg), ceftazidime (CAZ: 30 μg), cefepime (FEP: 30 μg), Cefoxitin (30 μg), meropenem (MER: 10 μg), gentamicin (GEN:10 μg), amikacin (30 μg) ciprofloxacin (CIP: 5 μg), norfloxacin (NOR: 10 μg) and sulfamethoxazole-trimethoprim (SXT: 3.75/1.25 μg). The antibiotic discs used were from Abtek Biologicals Ltd., Liverpool, United Kingdom product. An *Enterobacteriaceae* isolate was considered as MDR if it was non-susceptible to three or more drugs from different classes/groups of antibiotics [17].

### Screening for potential ESBL-producing isolate

The isolates that showed an inhibition zone size of ≤22 mm with ceftazidime (30 μg) and/or ≤ 27 mm with cefotaxime (30 μg) were considered as potential ESBL-producer (screening ESBL positive) and were selected for confirmation for ESBLs production using CDT as recommended by CLSI guideline [16].

### Confirmation of ESBLs with combination disc test

A disc of ceftazidime (30 μg), cefotaxime (30 μg) and cefepime (30 μg), and ceftazidime + clavulanic acid (30 μg/10 μg), cefotaxime (30 μg) + clavulanic acid (30 μg/10 μg) and cefepime (30 μg) + clavulanic acid (30 μg/10 μg) was placed at appropriate distance on a MHA plate inoculated with a bacterial suspension of 0.5 McFarland turbidity standards and incubated overnight (18–24 h) at 37 °C. Cefepime (30 μg) and cefepime (30 μg) + clavulanic acid (30 μg/10 μg) is EUCAST’s recommendation. An increase in the inhibition zone diameter of > 5 mm for a combination disc versus ceftazidime or cefotaxime disc alone was confirmed as ESBLs production [16, 18].

### Quality control and data quality assurance

Quality control for the new batch was performed using ATCC 25922 *E. coli* standard strain to check the quality of culture media and antibiotics disks. For the ESBL confirmatory test, *K. pneumoniae* ATCC® 700603 (ESBLs positive) and *E. coli* ATCC® 25922 (ESBLs negative) control strains were used to check the quality of the commercially purchased antibiotics disks and in-house prepared combination disks [16]. The data collection form was checked for its completeness and accuracy before recording the data. Culture and antibiotics susceptibility test results were recorded carefully before entry to SPSS software (version 20).

### Data entry and analysis

Data were entered and analyzed using SPSS software (version 20). Proportions and the actual number of ESBL-producing *Enterobacteriaceae* isolates were used to describe frequency outputs for categorical variables. The data were presented in table and graphs. Mean and standard deviation were used to describe continuous variables.

## Results

### Demographic characteristics of the patients

A total of 426 consecutive non-repetitive *Enterobacteriaceae* isolates were collected from the four microbiology laboratories from January 1 to May 30, 2017. During the study period, we obtained 150 isolates from International Clinical Laboratories (ICL), 118 isolates from Tikur Anbessa Specialized Hospital (TASH), 89 isolates from Clinical Bacteriology and Mycology National Reference Laboratory in Ethiopian Public Health Institute (EPHI), and 69 isolates from Yekatit 12 Medical College Hospital (YMCH). These isolates were identified from different clinical specimens: 272 from urine; 90 from blood; 40 from pus; 11 from body fluids; 6 from sputum; 3 from ear discharge; 2 from eye discharge; and 2 from cerebrospinal fluid (CSF) **(**Table 1**)**.

**Table 1:** Distribution of *Enterobacteriaceae* isolates against demographic characteristics, specimen types and bacteriology laboratory, Addis Ababa, Ethiopia between Jan to May 2017

Variables (Number)		Distribution *Enterobacteriaceae* isolate n (%)						
		*E. coli*	*K. pneumoniae*	*E. cloacae*	*Citrobacter species*	*K. oxytoca*	*K. ozaenae*	Other^a^ isolates
Gender	Male (190)	90 (47.4)	54 (28.4)	17 (8.9)	17 (8.9)	5 (2.6)	1 (0.5)	6 (3.1)
	Female (236)	138 (58.5)	49 (20.8)	5 (2.1%)	12 (5.0)	10 (4.2)	12 (5.1)	10 (4.2)
Age group	≤ 28 days (24)	3 (12.5)	18 (75.0)	0 (0.0)	0 (0.0)	0 (0.0)	3 (12.5)	0 (0.0)
	29 days- < 1 year (34)	9 (26.5)	21 (61.8)	0 (0.0)	1 (2.9)	2 (5.9)	0 (0.0)	1 (2.9)
	1- < 5 years (35)	16 (45.7)	9 (25.7)	1 (2.9)	1 (2.9)	1 (2.9)	0 (0.0)	7 (20.0)
	5- < 15 years (42)	18 (42.9)	14 (33.3)	2 (4.8)	1 (2.9)	3 (7.1)	0 (0.0)	4 (9.5)
	15- < 25 years (35)	14 (40.0)	5 (14.3)	4 (11.4)	5 (14.3)	4 (11.4)	3 (8.6)	0 (0.0)
	25- < 65 years (190)	119 (62.6)	30 (15.8)	7 (3.7)	18 (9.5)	4 (2.1)	5 (2.6)	7 (3.7)
	> 65 years (66)	49 (74.2)	6 (9.1)	4 (6.1)	4 (6.0)	1 (1.5)	2 (3.0)	0 (0.0)
Bacteriology laboratories	ICL (150)	109 (72.7)	9 (6.0)	6 (4.0)	13 (8.7)	3 (2.0)	6 (4.0)	4 (2.6)
	EPHI (89)	36 (40.4)	29 (32.6)	6 (6.7)	6 (6.7)	2 (2.2)	4 (4.5)	6 (6.7)
	TASH (118)	53 (44.9)	33 (28.0)	7 (5.9)	9 (7.6)	9 (7.6)	1 (0.8)	6 (5.1)
	YHMC (69)	30 (43.5)	32 (46.4)	3 (4.3)	1 (1.4)	1 (1.4)	2 (2.9)	0 (0.0)
Types of Specimen	Urine (272)	188 (69.1)	32 (11.8)	11 (4.0)	19 (7.0)	7 (2.6)	7 (2.6)	8 (2.9)
	Blood (90)	24 (26.7)	53 (58.9)	3 (3.3)	2 (2.2)	5 (5.6)	3 (3.3)	0 (0.0)
	Pus (40)	8 (20.0)	12 (30)	6 (15.0)	4 (10.0)	2 (5.0)	3 (7.5)	5 (12.5)
	Sputum (6)	1 (16.7)	2 (33.3)	1 (16.7)	2 (33.3)	0 (0.0)	0 (0.0)	0 (0.0)
	CSF (2)	0 (0.0)	2 (100.0)	0 (0.0)	0 (0.0)	0 (0.0)	0 (0.0)	0 (0.0)
	Body fluids (11)	5 (45.5)	2 (18.2)	1 (9.1)	1 (9.1)	1 (9.1)	0 (0.0)	0 (0.0)
	Ear & Eye discharge(5)	2 (40.0)	0 (0.0)	0 (0.0)	1 (20.0)	0 (0.0)	0 (0.0)	2 (40.0)
	Total (*N* = 426)	228 (53.5)	103 (24.1)	22 (5.2)	29 (6.8)	15 (3.5)	13 (3.1)	16 (3.8)

^a^Other isolates are *P. mirabilis*, *Providencia species, M. morganii* and *E. aerogenes*

Among the patients included in the study, 236 (54.4%) of the isolates were recovered from males and 190 (44.6%) from females. The most frequently isolate found among males were *E. coli* (47.4%) and *K. pneumoniae* (28.4%), and among females were *E. coli* (58.5%), *K. pneumoniae* (20.8%). The isolates were obtained from patients aged from 1 day to 91 years with the mean age of 32.6 years (standard deviation 25.6). Among all *Enterobacteriaceae* isolates, 58/426 (13.6%) were isolated from infants less than 1 year, 93/426 (21.8%) from children less than 5 years, and 135/426 (31.7%) from children less than 15 years of age (Table 1).

### Frequency of *Enterobacteriaceae* isolates

Among all *Enterobacteriaceae,* the most frequent isolates were *E. coli* (53.5%; 228/426) and *K. pneumoniae* (24.1%; 103/426). *E. coli* were predominantly isolated in urine (82.5%; 188/228) and in blood specimens (10.5%; 24/228). From the total *K. pneumoniae* isolate, 54.1% (53/103) were obtained from blood, 31.1% (32/103) from urine and 11.6% (12/103) wound/pus. Furthermore, from all *K. pneumonia* 50.5% (50/103) were isolated from children age less than 15 years (Table 1).

#### Antibiotics resistance pattern of *Enterobacteriaceae*

The antibiotics resistance pattern of *Enterobacteriaceae* isolated in different clinical specimens against 11 antibiotics is presented in Table 2**.** The highest resistance level was recorded for sulfamethoxazole-trimethoprim (77.0%), followed by amoxicillin with clavulanic acid (71.6%), cefotaxime (62.2%), cefepime (60.3%), ceftazidime (60.8%) and norfloxacin (58.8%). There was also a significant level of resistance to ciprofloxacin (46.3%), gentamycin (43.4%) and cefoxitin (25.1%). Lower resistance levels were observed against meropenem (5.2%) and amikacin (13.8%).

**Table 2:** Distribution of antibiotics resistance among *Enterobacteriaceae* isolates, Addis Ababa, Ethiopia between Jan to May 2017

Isolates (number)	Distribution of antibiotics resistance among *Enterobacteriaceae* isolates (n (%))										
	CTX	CAZ	CFP	FOX	MER	SXT	CPR	GEN	AMK	AMC	NOR^a^ N/Total
*E. coli* (*n* = 228)	125 (54.8)	121 (53.1)	122 (53.5)	50 (21.9)	8 (3.5)	177 (77.6)	146 (64.0)	76 (33.3)	27 (11.8)	160 (70.0)	121/188 (64.3)
*K. pneumoniae* (*n* = 103)	89 (86.4)	88 (85.4)	88 (85.4)	24 (23.3)	11 (10.7)	89 (86.4)	52 (50.5)	72 (70.0)	22 (21.3)	87 (85.4)	18/32 (56.2)
*E. cloacae* (n = 22)	12 (54.5)	11 (50.0)	12 (54.5)	14 (63.6)	0 (0.0)	14 (63.6)	9 (40.9)	9 (40.9)	0 (0.0)	16 (72.7)	3/11 (27.3)
*C. diversus* (*n* = 19)	12 (63.2)	12 (63.2)	12 (63.2)	5 (26.3)	1 (5.3)	15 (78.9)	12 (63.2)	10 (52.6)	0 (0.0)	13 (68.4)	6/6 (100)
*K. oxytoca* (*n* = 15)	8 (53.3)	8 (53.3)	7 (46.7)	2 (13.3)	1 (6.7)	9 (60.0)	5 (33.3)	5 (33.3)	1 (6.7)	8 (53.3)	3/7 (42.8)
*K. ozaenae* (*n* = 13)	7 (53.8)	7 53.8)	6 (46.2)	2 (15.4)	0 (0.0)	8 (61.5)	6 (46.2)	5 (38.5)	0 (0.0)	8 (61.5)	0/5 (0.0)
*Citrobacter. spps* (n = 10*)*	5 (50.0)	5 (50.0)	5 (50.0)	7 (70.0)	1 (10)	7 (70.0)	5 (50.0)	3 (30.0)	0 (0.0)	5 (50.0)	5/7 (71.4)
*Providencia spps* (*n* = 7)	3 (42.8)	3 (42.8)	3 (42.8)	2 (28.6)	0 (0.0)	3 (42.8)	2 (28.6)	1 (14.3)	0 (0.0)	4 (57.1)	2/4 (50.0)
*P. mirabilis* (*n* = 5)	1 (20)	1 (20)	1 (20)	0 (0.0)	0 (0.0)	3 (60)	2 (40)	1 (20)	0 (0.0)	0 (0.0)	0/1 (0)
*M. morganii* (n = 2)	2 (100)	2 (100)	2 (100)	1 (50)	0 (0)	2 (100)	1 (50)	1 (50)	0 (0)	2 (100)	0 (0)
*E. aerogens* (*n* = 2)	1 (50)	1 (50)	1 (50)	0 (0)	0 (0)	1 (50)	0 (50)	1 (50)	0 (0)	1 (50)	0/2 (0)
Total Resistance (N = 426)	265 (62.2)	257 (60.8)	259 (60.3)	107 (25.1)	22 (5.2)	324 (77.0)	240 (46.3)	185 (43.4)	59 (13.8)	305 (71.6)	160/272 (58.8)

Abbreviations: *CTX* cefotaxime, *CAZ* ceftazidime, *FOX* cefoxitin, *CFP* cefepime, *MER* meropenem, *CPR* ciprofloxacin, *NOR* norfloxacin, *SXT* sulfamethoxazole-trimethoprim, *GEN* gentamycin, *AMK* amikacin, *AMC* amoxicillin with calvulanic acid

^a^Norfloxacin antibiotics disks were used for isolates from urine specimen

*E. coli* showed the highest resistance to sulfamethoxazole-trimethoprim (77.6%) followed by amoxicillin-clavulanic acid (70.0%), norfloxacin (64.3%), and ciprofloxacin (64.0%). In addition, its resistance level to cefotaxime, cefepime, and ceftazidime was 54.8, 53.5, and 53.1% respectively. However, the lowest level of resistance was observed to MER (3.5%) and AMK (11.8%). In *K. pneumoniae,* high resistance was observed against cefotaxime (86.4%), cefepime (85.4%), ceftazidime (85.4%), amoxicillin-clavulanic acid (85.4%) and gentamicin (70.0%), with low resistance level to meropenem (10.7%) and amikacin (21.3%). **(**Table 2).

### Multi-drug resistant *Enterobacteriaceae*

**Fig. 1:**
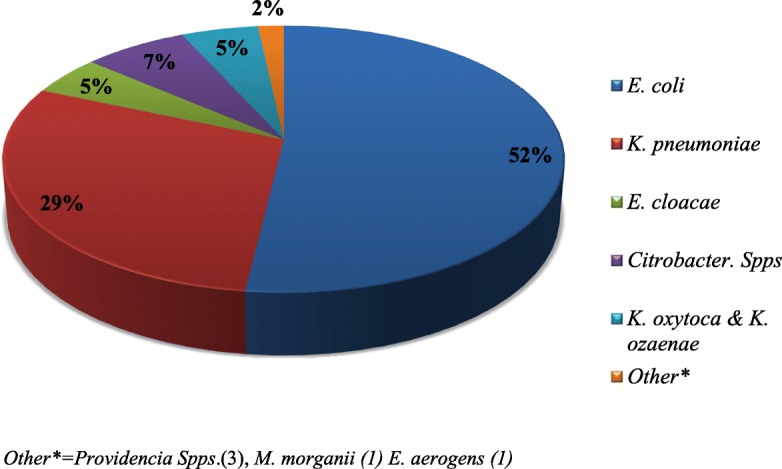
Distribution of major MDR isolate among the total MDR *Enterobacteriaceae between Jan to May 2017*

Overall, 68.3% (291/426) of the *Enterobacteriaceae* isolates were multi-drug resistant (MDR, non-susceptible to at least 3 antibiotics belonging to different antibiotics categories), among which *E. coli* and *K. pneumoniae* contributed to 35.0% (150/426) and 20% (85/426) of the observed MRD, respectively. We found that the highest MDR level was observed among *K. pneumoniae* isolates *(*83.5%, 86/103) followed by *citrobacter species* (68.9%, 20/29), *E. coli* (66.2%, 151/228), and *E. cloacae* (63.6%, 14/22). None of *P. mirabilis* was found to be MDR*.* Only 11.3% (48/426) of the *Enterobacteriaceae* were susceptible for all antibiotics tested in this study (Table 3). From all MDR *Enterobacteriaceae,* the predominant were *E. coli* (51.9%; 151/291) and *K. pneumoniae* (29.6%; 86/921) **(**Fig. 1).

**Table 3:** Multidrug resistance level of *Enterobacteriaceae* to different classes of antibiotics, Addis Ababa, Ethiopia between Jan to May 2017

Isolates (number)	Level of antibiotics resistance ((number (%))								Total MDR-E (>R3)
	RO	R1	R2	R3	R4	R5	R6	R7	
*E.coli* (228)	20 (8.8)	28 (12.3)	29 (12.7)	34 (14.9)	44 (19.3)	50 (21.9)	16 (7.0)	5 (2.2)	149 (65.3)
*K.pneumoniae* (103)	6 (5.8)	5 (4.8)	7 (6.8)	10 (9.7)	22 (21.3)	33 (32.0)	11 (10.7)	9 (8.7)	85 (82.5)
*E.cloacae* (22)	4 (18.2)	2 (9.1)	2 (9.1)	3 (13.6)	2 (9.1)	3 (13.6)	6 (27.3)	0 (0.0)	14 (63.6)
*C.diversus* (19)	3 (15.8)	2 (10.5)	1 (5.3)	1 (5.3)	3 (15.8)	5 (26.3)	3 (15.8)	1 (5.3)	13 (68.4)
*K.oxytoca* (15)	5 (33.3)	2 (13.3)	1 (6.7)	0 (0.0)	2 (13.3)	4 (26.7)	0 (0.0)	1 (6.7)	7 (46.6)
*K.ozaenae* (13)	1 (7.7)	3 (23.1)	1 (7.7)	2 (15.4)	5 (38.5)	1 (7.7)	0 (0.0)	0 (0.0)	8 (61.5)
*Citrobacter. Spps*(10)	3 (30.0)	0 (0.0)	0 (0.0)	3 (30.0)	0 (0.0)	2 (20.0)	1 (10.0)	1 (10.0)	7 (70)
*Providencia Spps*.(7)	3 (42.8)	0 (0.0)	1 (24.3)	0 (0.0)	2 (28.5)	1 (24.3)	0 (0.0)	0 (0.0)	3 (42.8)
*P. mirabilis* (5)	2 (40.0)	1 (20.0)	2 (20.0)	0 (0.0)	0 (0.0)	0 (0.0)	0 (0.0)	0 (0.0)	0 (0.0)
*M. morganii* (2)	0 (0.0)	0 (0.0)	0 (0.0)	1 (50.0)	0 (0.0)	0 (0.0)	1 (50.0)	0 (0.0)	1 (50.0)
291 (68.3)	1 (50.0)	0 (0.0)	0 (0.0)	0 (0.0)	1 (50.0)	0 (0.0)	0 (0.0)	0 (0.0)	1 (50.0)
Total(N = 426)	48 (11.3)	43 (10.1)	43 (10.1)	54 (12.7)	81 (19.0)	101 (23.7)	38 (8.9)	17 (4.0)	291 (68.3)

Abbreviations: R0 stands for resistance for zero antibiotics; R1 stands for resistance to one drug, R2 stands for resistance to two drugs and so on; and ≥ R3 stands for resistance to 3 or more antibiotics from different classes; MDR-E stands for multi-drug resistant *Enterobacteriaceae*

#### Magnitude of ESBL producing *Enterobacteriaceae*

Of all the *Enterobacteriaceae* isolates*,* 62.2% (265/426) were positive for the screening test of ESBL production as measured with cefotaxime zone of inhibition ≤27 mm and ceftazidime zone of inhibition ≤22 mm. Using the combination disk test, we confirmed that 92.8% (246/265) of the suspected isolates were able to produce ESBL resulting in an overall ESBLs positivity of 57.7% (246/426) (Table 4). From all the isolate, *E. coli* accounted 27.9% (119/426), *K. pneumoniae* 19.0% (81/426), and other *Enterobacteriaceae* 10.8% (46/426).

**Table 4:** Distribution of major ESBL-producing *Enterobacteriaceae* and MDR in four microbiology laboratory/hospitals in Addis Ababa, Ethiopia between Jan to May 2017

Isolate collection Site	ESBL-producing *Enterobacteriaceae* (n (%))	Major ESBL-producing *Enterobacteriaceae*			MDR *Enterobacteriaceae* (n (%))
		*E. coli* (n (%))	*K. pneumoniae* (n (%))	*E. coli* and *K. pneumoniae* (n (%))	
TASH	71.5 (84/118)	64.2 (34/53)	84.8 (28/33)	72.1 (62/86)	79.7 (94/118)
YHMC	68.1 (47/69)	53.3 (16/30)	84.4 (27/32)	69.3 (43/62)	71.0 (49/69)
EPHI	66.3 (59/89)	75.0 (27/36)	72.4 (21/29)	73.8 (48/65)	70.8 (63/89)
ICL	37.3 (56/150)	38.5 (42/109)	55.6 (5/9)	39.8% (47/118)	56.7 (85/150)
Total	57.7 (246/426)	52.2 (119/228)	78.6 (81/103)	60.4 (200/331)	68.3 (291/426)

**Fig. 2:**
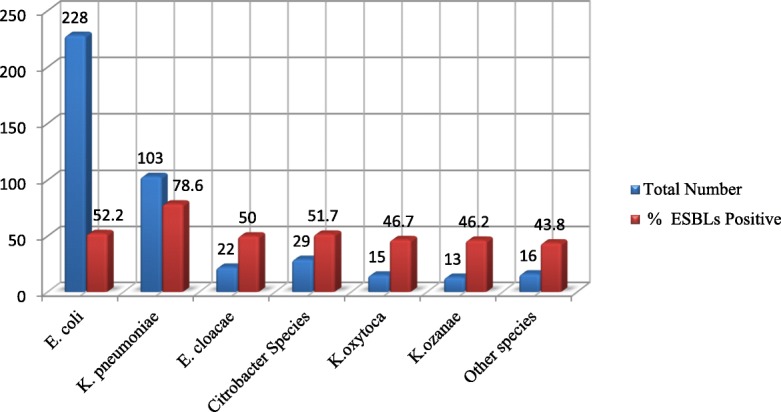
Frequency of ESBLs producing *Enterobacteriaceae* species between Jan to May 2017

The distribution of ESBL producers varied among different species of *Enterobacteriaceae*. The highest intra-species frequency of ESBL production was observed among *K. pneumoniae* 78.6% (81/103) followed by *E. coli and Citrobacter species* with 52.2% (119/228) and 51.7% (15/29), respectively ***(***Fig. *2****)*.** The lowest intra-species ESBL production was observed in *P. mirabilis with 20% (1/5) proportion.*

Regarding ESBL-producing *Enterobacteriaceae* distribution across age groups, a higher proportion was observed among isolates from patients less than 1 year (86.2%), < 28 days (87.5%), and 5 to < 15 years (69.0%) compared with other age groups. The total proportion of ESBL-producing *Enterobacteriaceae* among children < 15 years was 74.1% (100/135).

#### Distribution of MDR and ESBL-producing *Enterobacteriaceae*

The magnitude of ESBL-producing *Enterobacteriaceae* was different in the four microbiology laboratories. The magnitude was highest in TASH (71.5%; 84/118) followed by YHMC (68.1%; 47/69) and EPHI (66.3%; 59/89), and lowest in ICL (37.3%; 56/150). In all laboratories, the highest ESBL production was observed among *K. pneumoniae* (78.6%; 81/103)*.* Distribution of MDR *Enterobacteriaceae* and major ESBL-producing *Enterobacteriaceae* at the four microbiology laboratories is presented in Table 4.

### Distribution of ESBL-producing *Enterobacteriaceae* with their MDR level among different clinical specimens

From all specimens included in this study, the highest magnitude of ESBL-producing *Enterobacteriaceae* (84.4%; 76/90) and MDR (83.3%; 75/90) was found in blood. In the urine specimen, the extent of ESBL-producing *Enterobacteriaceae* and MDR were 50.7% (138/272) and 66.5% (181/272), respectively (Table 5). Of all ESBL-producing *Enterobacteriaceae*, 96.3% (237/246) were MDR, whereas only 30% (54/180) of the non–ESBL producers were MDR. There was a significant correlation (Pearson correlation of 0.759, *p*-value of 0.01) between ESBL production and MDR *Enterobacteriaceae*. Binary logistic regression or bivariate analysis also showed that being an ESBL producer has statistically significant association with MDR (*P* < 0.001). That is, the odds of being MDR were 61.4 times (95% CI COR = 29.37 to 128.53) more likely among ESBL-producing *Enterobacteriaceae* than non-ESBL isolates.

**Table 5:** Distribution of ESBL-producing *Enterobacteriaceae* with their MDR level in different clinical specimens, Addis Ababa between Jan to May 2017

Specimens (number)	MDR-E n (%)		ESBLs test result n (%)		
	YES	NO	POS	NEG	Non-ESBL Suspicious
Urine (272)	181 (66.5)	91 (33.5)	138 (50.7)	14 (5.1)	120 (44.1)
Blood (90)	75 (83.3)	15 (16.7)	76 (84.4)	3 (3.3)	11 (12.2)
Wound or Pus (40)	23 (57.5)	17 (42.5)	21 (52.5)	2 (5.0)	17 (42.5)
Other specimens^a^ (24)	12 (50.0)	12 (50.0)	11 (45.8)	0 (0.0)	13 (44.2)
Total (*N* = 426)	291 (68.3)	135 (31.7)	246 (57.7)	19 (4.5)	161 (37.8)

^a^ Other specimens, CSF & other body fluids, sputum, ear and eye discharge

#### Antibiotics susceptibility pattern of ESBLs-E to potentially active drugs

**Fig. 3:**
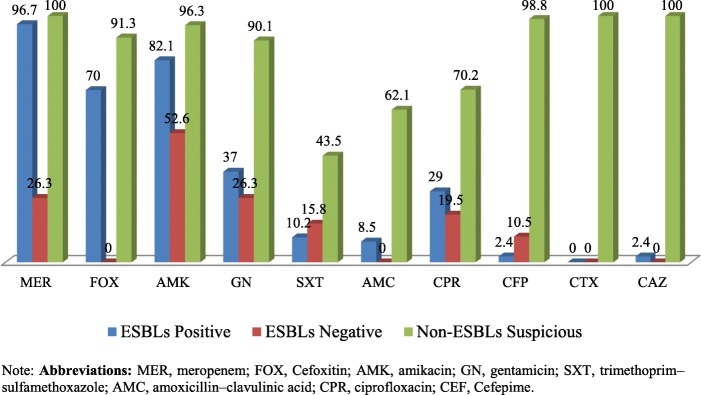
Antibiotics susceptibility pattern of ESBLs-positive, ESBLs-negative and Non-ESBLs Suspicious *Enterobacteriaceae between Jan to May 2017*

The most active drugs for ESBL-producing isolates were meropenem, amikacin, and cefoxitin, with susceptibility results of 96.7, 82.1, and 70%, respectively. Moreover, 37, 29, and 10.2% of ESBL-producing isolates were susceptible to gentamicin, ciprofloxacin, and cotrimoxazole, respectively. Non-ESBL-suspicious isolates were 100, 96.3, and 91.3% sensitive to meropenem, amikacin, and cefoxitin, respectively. Furthermore, gentamicin and ciprofloxacin remained active against 90.1 and 70.2% respectively of non-ESBL-suspicious *Enterobacteriaceae*. The antibiotic susceptibility of ESBL confirmatory test positive, ESBL screening test positive-producing, and non-ESBL-suspicious (screening negative) *Enterobacteriaceae* is displayed in Fig. 3.

## Discussions

ESBL-producing *Enterobacteriaceae* have become a serious worldwide problem. Dissemination of ESBLs compromises the activity of broad-spectrum antibiotics creating major therapeutic difficulties with a significant impact on the outcomes for patients [19].

### Prevalence of ESBL-producing *Enterobacteriaceae*

In the present study, the magnitude of ESBL-producing *Enterobacteriaceae* was 57.7%, which is higher than magnitudes reported by previous researchers in Ethiopia: 38.4% in Jimma by Siraj and his colleagues [20], 36% in Jimma by Mulualem Y and his colleagues [21], 33.3% in Harar [22] and 25% in Adama [23]. The emergence of ESBL-producing *Enterobacteriaceae* in higher magnitude in Addis Ababa emphasizes the need to implement strong infection control strategies.

The magnitude of ESBL-producing *Enterobacteriaceae* (57.7%) in our study was comparable with a studies in Bahir-Dar-Ethiopia (57.6%) [24], Burkina Faso (58.0%) [25], Uganda (62.0%) [26], Ghana (49.3%) [27], and Karnataka-India (57.5%) [28]. One of the most important factors in the emergence of ESBLs production is the selective pressure caused by the use of 3rd generation cephalosporins [29, 30]. Lack of antibiotic surveillance, antibiotics misuse, and weak infection control measures may also contribute to the high magnitude of ESBL.

Compared with the present study, ESBL-producing *Enterobacteriaceae* prevalence in Europe is lower; 0.7% in Austria and 23.8% in Turkey [31], and 6.3% in Italy [32]. The difference might be due to infection control strategies in those countries. Moreover, our finding is higher than levels seen in some non-European countries, such as Egypt (16%) [33] and Nepal (24.4%) [34]. The difference may be due to the study participant and method difference.

The predominant ESBL-producing isolates in this study, *K. pneumoniae* (78.6%) and *E. coli* (52.2%) were in agreement with studies done in Bahir-Dar, Ethiopia: (*K. pneumoniae* 69.8%, *E. coli* 58.2%) [24], Jimma, Ethiopia (*K. pneumoniae* 70.4%, *E. coli* 28.2%) [20], and Uganda: (*K. pneumoniae* 72.7%, *E. coli* 58.1) [26]. However, *E. coli* was a predominant ESBL producer compared with *K. pneumoniae* in studies in Adama, Ethiopia (*E. coli* 51.5%, *K. pneumoniae* 11.5%) [23], Burkina Faso (*E. coli* 67.5%, *K. pneumoniae* 26%) [25], India (*E. coli* 61.4%, *K. pneumoniae* 46.2%) [28] and Central India (*E. coli* 50.14%, *K. pneumoniae* 48.27%) [35].

The proportion of ESBL-producing *Enterobacteriaceae* among children under 15 years (74.1%) was in agreement with the previous studies done in Addis Ababa TASH (78.57%) [36], Tertiary Care Hospital of North-West India (66.7%) [37] and in rural Ghana (68%) (38) [38]. However, our finding was higher compared to a study conducted in Burkina Faso (50.8%) [25].

### Distribution of ESBL-producing *Enterobacteriaceae* among different specimens

In our study ESBL-producing *Enterobacteriaceae* were found predominantly in blood specimens (84.4%, 76/90) followed by wound/pus specimens (52.5%, 21/40), urine (50.7, 138/272) and other specimens (CSF & other body fluids, sputum, ear and eye discharge) (45.8%, 11/24). Other investigator also reported blood as a major source of ESBL-producers in Bahir-Dar Dar (84.8% in blood, 72.7% in open wound swabs) [24], Burkina Faso (75% in blood) [25], Iran (87.8% in blood, 48.5% in urine) [39], North West India (79.2.0% in blood) [37] and again in India (66.67% in blood, 54.67% in urine) [40]. This indicates that ESBL-producing *Enterobacteriaceae* are becoming a serious problem in the treatment of invasive bacterial infections. However, in other studies urine was the major source of ESBL-producers: central India (52.28% in urine) [35], Uganda (64.9% in urine, 47.4% in pus) [26], Bangladesh (70.4% in urine, 16.5% in blood) [41]. The difference might be attributed to the difference in the study participants, risk factors or extent of antibiotics use.

### Antibiotics susceptibility pattern of ESBL-producing *Enterobacteriaceae*

In this study, ESBL-producing isolates were found to be susceptible primarily to meropenem (96.7%), amikacin (82.1%), and cefoxitin (70%). This was in close agreement with studies done in Ghana (meropenem 100%) [27], central India (meropenem 87.5%, amikacin 83.92%) [35], Jimma, Ethiopia (amikacin 83.7%) [20], and India (meropenem 94.0%, amikacin 82.6%) [42]. The results indicate that these antibiotics were the most active treatment of choice for ESBL-producing *Enterobacteriaceae.*

In the present study, the levels of co-resistance within different classes of antibiotics among the ESBL-producing *Enterobacteriaceae* were significantly higher for most antibiotics tested. Of ESBL-producers, 63% were non-susceptible to gentamicin, 89.8% to trimethoprim-sulfamethoxazole, 69% to ciprofloxacin, 97.6% to cefepime, and 91.5% to amoxicillin-clavulanic acid. Our finding is comparable with the study conducted in Israel, which showed that 75% of ESBL-producer isolates were non-susceptible to gentamicin, 70% to trimethoprim-sulfamethoxazole and 59% to ciprofloxacin [9], and also comparable with studies in Burkina Faso (45% to trimethoprim-sulfamethoxazole, 89% to gentamicin, 80% to ciprofloxacin) [25], Ghana (92.6% to trimethoprim-sulfamethoxazole, 91.2% to gentamicin, 41.1% to ciprofloxacin) [27], Nepal (90.7% to ciprofloxacin, 90.4% to trimethoprim-sulfamethoxazole, 63.12% to gentamicin) [34], and central India (50% to gentamicin, 87.5% to ciprofloxacin, 94.6% to trimethoprim-sulfamethoxazole) [25]. These findings indicate that ESBL-producing *Enterobacteriaceae* were the major cause of resistance to various antibiotics classes, as these bacteria are typically nosocomial.

### Antibiotics resistance pattern among all *Enterobacteriaceae* isolates

In the present study, high resistance was observed to sulfamethoxazole-trimethoprim (77.0%) followed by amoxicillin with clavulanic acid (71.6%), cefotaxime (62.2%), ceftazidime (60.8%), cefepime (60.3), norfloxacin (58.8%), ciprofloxacin (46.3%) and gentamycin (43.4%). The results of our study are in line with the findings of studies conducted in Iran (sulfamethoxazole-trimethoprim 94%, gentamycin 57.8%, ceftazidime 73%, ciprofloxacin 55.5% [39], Nepal (sulfamethoxazole-trimethoprim 62.1%, ceftazidime 83.2%, cefotaxime 74.7%, ciprofloxacin 61.1%, norfloxacin 64.2%) [43], and Sierra Leone (ceftazidime 62.9%, ciprofloxacin 74.2%, gentamycin 74.3%) [44]. This indicates that resistance rate to the commercially available as well as commonly used drugs is becoming alarming.

The resistance level of *Escherichia coli* to sulfamethoxazole-trimethoprim (77.6%), amoxicillin with clavulanic acid (70.0%), norfloxacin (64.3%) and ciprofloxacin (64.0%) in our study was concordant with studies conducted in Dessie, Ethiopia (sulfamethoxazole-trimethoprim 65.1%) [45], Gondar, Ethiopia (sulfamethoxazole-trimethoprim 78.3%) [46], Tanzania (sulfamethoxazole-trimethoprim 76%) [47], Khartoum, Sudan (sulfamethoxazole-trimethoprim 88.3%, amoxicillin-clavulanic acid 51.4%, ciprofloxacin 58.4%) [48]. On the other hand, our findings were lower than the finding in Iran (sulfamethoxazole-trimethoprim 92.8%) [39] and Equatorial Guinea (sulfamethoxazole-trimethoprim 95%, amoxicillin-clavulanic acid 88.4%, ciprofloxacin 59.8%) [49]. This shows that the treatment option for the most common cause of nosocomial pathogen is becoming lower.

In *K. pneumoniae* the highest level of resistance was observed against sulfamethoxazole-trimethoprim (86.4%) cefotaxime (86.4%), cefepime (85.4%), ceftazidime (85.4%), amoxicillin-clavulanic acid (85.4%), gentamicin (70.0%) and ciprofloxacin (50.5%). There were also similar findings from studies conducted in Iran (sulfamethoxazole-trimethoprim 91.4%, ceftazidime 91.4%, and gentamicin 82.8%) [39], Sierra Leone (ciprofloxacin 73.4%, gentamicin 60%) [44], Equatorial Guinea (sulfamethoxazole-trimethoprim 100%, amoxicillin-clavulanic acid 96.6%, gentamicin 86.2%, ciprofloxacin 87.5%) [49]. The high resistance rate of *Enterobacteriaceae* to the antibiotics may be due to the misuse or overuse of the antibiotics coupled with weak infection control measures [19]. The high resistance rate of *K. pneumoniae* alerts the health care system to work hard on the health facilities infection control.

### Multi-drug resistance among *Enterobacteriaceae*

In the present study, the overall magnitude of MDR among all *Enterobacteriaceae* isolate (68.3%) was fairly similar with a study done in Dessie, Ethiopia (74.6%) [45], Gondar, Ethiopia (68%) [50], and Nepal (64.04%) [51]. The higher proportion of MDR limits the treatment option for hospital-acquired infections caused by *Enterobacteriaceae.* On the other hand, our result was lower than findings from other studies in Gondar, Ethiopia (93.5 and 87.4%) [46, 52], Bahir-Dar (93.1%) [53], Nepal (96.84%) [43], and Sierra Leone (85.7%) [44]. The difference in magnitude of MDR isolates might be due to the selection of antibiotic from a different class, the definition for MDR, study period and specimen type, and the difference in the study population.

There was an intra-species difference in MDR level. The present study showed that the level of MDR in *K. pneumoniae* (82.5%) and *E. coli* (65.3%) was comparable with studies conducted in Equatorial Guinea (*E. coli* 74.4%) [49], Sierra Leone (*K. pneumoniae* 73.3%, *E. coli* 61.5%) [44]. However, our result is lower than studies conducted in Gondar, Ethiopia (*K. pneumoniae* 95.6%, *E. coli* 92.9% [52], Khartoum, Sudan (*E. coli* 92.2%) [48], and Equatorial Guinea (*K. pneumoniae* 91.7%) [49]. The MDR level among *E. coli* (50.2%) in Dessie, Ethiopia is lower than our study [45]. The difference in MDR level among *K. pneumoniae and E. coli* in our study might be due to most *K. pneumoniae* being isolated from blood specimens collected from hospital inpatients.

In this study 237 (96.3%) of the ESBL-producers were MDR strains, whereas only 54 (30%) of the non-ESBL-producers were MDR strains. The ESBL-producing isolates had increased resistance compared with non–ESBL-producers indicating that MDR is expected to be more common in ESBL-producing bacteria.

### Strength of the study

This is the first study done at multiple health facilities on the magnitude of ESBL-producing *Enterobacteriaceae* in Addis Ababa, Ethiopia. This multi-centered study can reveal the extent of distribution of ESBLs and MDR among *Enterobacteriaceae* and the degree of resistance to other non-beta-lactam antibiotics. The magnitude of ESBLs and MDR in the city was done in a relatively larger number of specimens and isolates than in earlier studies.

### Limitation of the study

- Although combinations of aminoglycosides and fluoroquinolones were tested, other beta-lactams and beta-lactamase inhibitors, such as tigacycline, colistin, and piperacillin/tazobactam, were not tested, as they were beyond the scope of this study.
- We are unable to see possible risk factors, certain clinical features and the outcome of the patients infected with ESBL-producing or MDR bacteria, due to lack of adequate resource.
- Although most of the study isolates were collected from inpatients, the exact number of nosocomial versus community-acquired bacteria were not differentiated.
- The isolates were collected from four bacteriology laboratories in Addis Ababa, but the results may not be applied to the entire city or country.

### Conclusion and recommendation

There was a high prevalence of ESBL-producing *Enterobacteriaceae* and MDR isolates. The majority of ESBL-producing isolates were found primarily in blood and urine specimens. The most frequent ESBL-producing *Enterobacteriaceae* were *K. pneumoniae* and *E. coli*. A higher level of resistance to multiple classes of antibiotics was observed among ESBL producers compared with non-ESBL producers. The better options for the treatment of ESBL-producing *Enterobacteriaceae* are meropenem, amikacin, and cefoxitin. ESBL-producing isolates showed a high rate of resistance to ciprofloxacin, cefepime, cotrimoxazole, and gentamicin compared with non-ESBL producers. The rise of MDR and ESBLs necessitates the strengthening of clinical bacteriology research and the diagnostic capacity of laboratory professionals for the detection and surveillance of antibiotic resistance. We recommend routine screening of ESBLs production of *Enterobacteriaceae* along with strong infection prevention strategies.

## Abbreviations

CDT: Combination disk test
CLSI: Clinical and Laboratory Standards Institute
EPHI: Ethiopian Public Health Institute
ESBL-E: Extended-spectrum beta-lactamases *Enterobacteriaceae*
ESBLs: Extended-spectrum beta-lactamases
ICL: International Clinical Laboratories
MDR: Multidrug Resistant
TASH: Tikur Anbessa Specialized Hospital
YHMC: Yekatit 12 Hospital Medical College
